# Authors' reply

**DOI:** 10.4103/0019-5413.43403

**Published:** 2008

**Authors:** Sanjiv Kumar Singh Marya, Rajiv Thukral, Chandeep Singh

**Affiliations:** *Max Institute of Orthopedics and Joint Replacement, Max Super Specialty Hospitals, 1, Press Enclave Road, Saket, New Delhi - 110 017, India*

Sir,

In response to the reader's comments, we would like to state that we completely agree with his observations that conventional teaching regarding the use of cementless femoral stems has been as per Engh *et al*.^1^ and Dorr *et al*.^2^

However, we observed a far higher morbidity in the form of acute hypotension and pulmonary embolism following the implantation of cemented stems in elderly patients. After an unfortunate incidence wherein a 78-year-old male patient who underwent cemented bipolar hemiarthroplasty (for fracture neck of femur) went into acute hypotension crisis, and who, despite best intensive care, could not be revived, the senior authors decided to perform cementless arthroplasty in elderly patients who have greater number of comorbid factors, and obviously poorer bone stock. This has been supported by other researchers in their reports.^3^–^6^

We have found that these patients do extremely well, without any unpleasant situations on table or immediately postoperative, thereby convincing us to recommend cementless stems for these very elderly patients even with poor bone stock.
